# Activity-Dependent Fluctuations in Interstitial [K^+^]: Investigations Using Ion-Sensitive Microelectrodes

**DOI:** 10.3390/molecules28020523

**Published:** 2023-01-05

**Authors:** Hana Beswick-Jones, Amy J. Hopper, Angus M. Brown

**Affiliations:** 1 School of Life Sciences, University of Nottingham, Nottingham NG7 2UH, UK; 2 Department of Neurology, University of Washington, Seattle, WA 98105, USA

**Keywords:** astrocyte, action potential, buffering, interstitial

## Abstract

In the course of action potential firing, all axons and neurons release K^+^ from the intra- cellular compartment into the interstitial space to counteract the depolarizing effect of Na^+^ influx, which restores the resting membrane potential. This efflux of K^+^ from axons results in K^+^ accumulation in the interstitial space, causing depolarization of the K^+^ reversal potential (E_K_), which can prevent subsequent action potentials. To ensure optimal neuronal function, the K^+^ is buffered by astrocytes, an energy-dependent process, which acts as a sink for interstitial K^+^, absorbing it at regions of high concentration and distributing it through the syncytium for release in distant regions. Pathological processes in which energy production is compromised, such as anoxia, ischemia, epilepsy and spreading depression, can lead to excessive interstitial K^+^ accumulation, disrupting sensitive trans-membrane ion gradients and attenuating neuronal activity. The changes that occur in interstitial [K^+^] resulting from both physiological and pathological processes can be monitored accurately in real time using K^+^-sensitive microelectrodes, an invaluable tool in electrophysiological studies.

## 1. Introduction

The introduction of invertebrate giant axons as the model of choice for electrophysiological studies in the mid-1930s [1] not only precipitated the experiments required to quantify the permeability changes associated with the action potential [2] but also allowed the investigation of trans-membrane ion fluxes that underlie neuronal activity [3]. The large diameter of these axons (the squid *Loligo* for the former experiments, the cuttlefish *Sepia* for the latter) introduced an intracellular electrode into the lumen of the axon to record the trans-membrane voltage, permitting the accurate quantification of the key features of the action potential profile (resting membrane potential, peak amplitude and after-hyperpolarization (AHP)) and also allowed estimations [4,5] and direct measurements [6,7] of compartmental changes in ion concentrations that result from neural activity. 

As a result of these classic experiments from the golden age of electrophysiology, a holistic picture emerged of the symbiotic relationship between neural elements and astrocytes with regard to regulating interstitial K^+^. Neuronal firing leads to K^+^ efflux from axons and extracellular accumulation, the degree of accumulation directly proportional to the firing frequency. The delicate balance of trans-membrane ion gradients governs firing activity, and thus maintaining this balance is of vital importance for optimal neuronal firing. Astrocytes act as a sink for interstitial K^+^, rapidly clearing it from the interstitial fluid and releasing it in quiescent locations with low interstitial [K^+^] ([K^+^]_o_) The advent of K^+^-sensitive microelectrodes allowed accurate recordings of real time fluctuations in [K^+^]_o_ that could be correlated with sharp electrode recordings of firing activity. A picture emerged of the efficient buffering of interstitial K^+^ in response to a brief rapid burst of action potentials in both grey and white matter.

The aim of this article is to review the results that have been obtained using K^+-^sensitive microelectrodes to record interstitial [K^+^] in central nervous tissue in response to a variety of physiological and pathological conditions.

## 2. Initial Studies Suggested ion Movements Underlie the Action Potential

The recording of the first intracellular action potential in 1939 [8] was followed 10 years later by a detailed study of the effects of altering Na^+^ or K^+^ in the seawater bathing the axon on the action potential profile. These experiments led to assumptions regarding trans-membrane ion movements that underlie the action potential, comparing experimental measures of the action potential waveform with the Nernstian predictions of reversal potentials that provided convincing evidence that the influx of Na^+^ expedited the rising phase of the action potential, whereas K^+^ efflux underlay the repolarising phase and AHP [9]. The subsequent Hodgkin and Huxley work estimated the charge movement across the membrane associated with a single action potential [10]. The value was in the order of picomoles per action potential, leading initially to the obvious assumption that such a small number of ions moving would have no effect on trans-membrane gradients. However, Hodgkin and Huxley were aware that as a result of the substantial electrical activity that occurs with trains of action potentials, changes to ion gradients would occur [2], but they carried out no direct work on this issue. Richard Keynes was a PhD student under the tutelage of Hodgkin in the late 1940s at Cambridge. Keynes was interested in electrical activity but decided to tackle the issue not by using the conventional electrophysiological equipment used by Hodgkin and Huxley [11] but rather by using radioactive tracers to measure the ion movements that result from electrical activity [7]. This technique offered the obvious advantage that it directly measured influx and efflux independently, whereas the measures of current used by Hodgkin and Huxley were an aggregate of influx and efflux, which provided limited information. Keynes showed that action potentials were indeed the result of an early Na^+^ influx followed by a delayed K^+^ efflux [7]. This paper was followed by several others, which all described the same phenomenon that K^+^ left the axon during the repolarising phase of the action potential [5]. An important conceptual advancement was made when Hodgkin and Huxley, using electrical techniques that linked changes in membrane capacitance to K^+^ efflux, demonstrated that the quantity of K^+^ efflux was directly proportional to the stimulus frequency [4], indicating a constant amount of K^+^ leaving the axon during each action potential.

The exit route of K^+^ during an action potential from squid axon was named the ‘delayed rectifier’ by Hodgkin and Huxley [12], since its kinetics were slower that the Na^+^ permeability pathway, and it favoured passing outward current over inward current. The advances in knowledge of the increasingly diverse population of excitable cells prompted questions of whether the Hodgkin and Huxley scheme was universally applicable. In the 1970s, the wide diversity of K^+^ channels became apparent, allowing their broad classification into the following families based on amino acid homology: delayed rectifiers, inward rectifiers and two-pore-domain channels [13]. Delayed rectifiers can be relatively fast to limit action potential duration, which occurs in unmyelinated axons, motor neurones and vertebrate fast skeletal muscle. Slow delayed rectifier channels serve a different purpose, such as in cardiac cells, which have long-duration action potentials, as the kinetics of the channels are slower [14]. The I_A_ current is radically different from the delayed rectifier described by Hodgkin and Huxley as it slowly inactivates [15], passing outward current that limits the depolarisation of the membrane and decreases firing frequency [16]. Inward rectifier channels tend to pass very small currents during depolarisation and are present in cardiac cells, as well as muscle and egg. They are unusual in that they open during hyperpolarisation and pass significant inward current at potential that is more hyperpolarised than E_K_. However, under physiological conditions, the function of the channels is to pass a small outward current at rest, but upon depolarisation, other currents activate, depolarising the membrane when the inward rectifiers close and no longer influence the membrane potential [17]. The role of two-pore-domain leak channels is discussed in Section 7.

The influence of the K^+^ efflux from axons depends on the dimensions of the interstitial space. As shown by Frankenhaueser and Hodgkin, when K^+^ leaving the axon is concentrated in a small volume it has a significant effect on the axon membrane potential, since the rise in [K^+^]_o_ depolarises E_K_, and hence depolarises the axon membrane potential [18]. In the sciatic nerve, however, the interstitial volume is large; thus, any axonal K^+^ efflux is diluted and relatively modest elevations in [K^+^]_o_ are recorded as a result of nerve stimulus [19].

The patch clamp technique, invented by Neher and Sakmann, can be utilised in a variety of modes [20]. However, of relevance to this review is the cell-attached mode, which directly measures the currents that cross the membrane through single ion channels. This technique can be used to quantify the amount of K^+^ loss from axons, since ions move through a channel at a fixed rate, and hence the constant amplitude of currents recorded in cell attached mode [13]. In voltage clamp mode, this rate is easy to measure but is far more difficult in current clamp mode, which more closely resembles physiological activity. However, Ohm’s law limits channel conductance and a routine assumption applied is that the same amount of K^+^ leaves an axon during each action potential. This would only stop applying if [K^+^]_o_ were to significantly increase.

## 3. Estimates of Interstitial K^+^ Accumulation following Action Potential Firing

**Figure 1:**
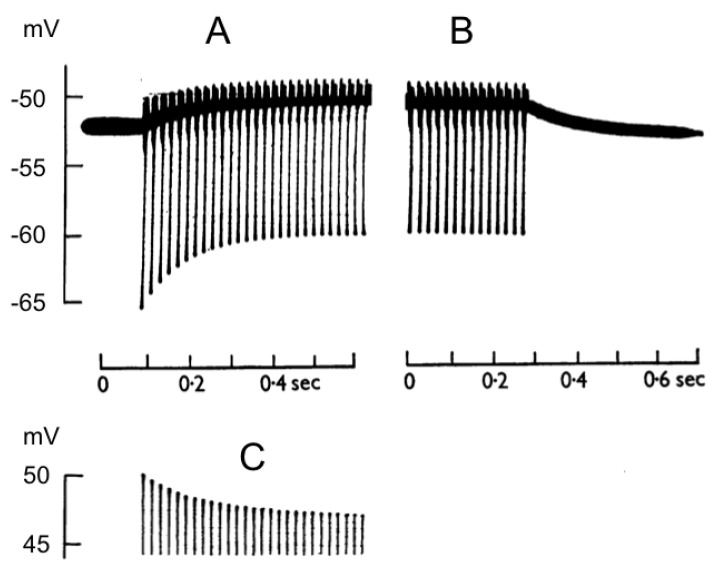
Recording of a train of action potentials evoked at 50 Hz, which result in (**A**) a decrease in the peak amplitude of later action potentials, (**B**) development of a depolarisation of the membrane potential and a decrease in the AHP amplitude. (**C**) On cessation of the stimulus, the membrane potential returns to rest (adapted from Ref. [18]).

In 1955, Hodgkin and Keynes published two influential papers. The first demonstrated that an adenosine tri-phosphate (ATP)-dependent process was required to re-equilibrate ions after activity; if the axons were poisoned, the ion gradients dissipated [21]. The other paper predicted the presence of ion channels and the sequential, single file movement of K^+^ ions through these channels [3]. There were extremely important consequences from these papers. The first was that the nature of ion movement through channels limited the number of ions that could cross the membrane, which provided estimates of single channel conductance, important in the development of the patch clamp method. The second was that the amount of energy required to re-equilibrate ions was dependent on the degree of neuronal activity [21], such that high-frequency firing resulted in large ion movements, which required more energy. An implication of this was that provided sufficient energy was available, neuronal activity did not disturb the ionic equilibrium and firing could be sustained indefinitely without any degradation of the signal. The consequences of the sequential firing of action potentials were investigated in the mid-1950s in the wake of the classic series of Hodgkin and Huxley papers. It is interesting to read the introduction to the Frankenhaeuser and Hodgkin paper, which is forthright about the serendipitous nature of the experiments [18]. They used the squid giant axon to investigate what they initially assumed to be calcium effects on firing but soon realised was due to extra-axonal K^+^ accumulation, resulting from action potentials. They found that the early spikes influenced the profile of later spikes in a train of action potentials, the main effects being the development of a depolarising after potential and a decrease in the AHP (Figure 1). Both effects were dependent on the stimulus frequency; the higher the frequency, the shorter the rate constant for the effect. The only technique available to them was the intracellular recording of membrane potential, but Frankenhaeuser and Hodgkin resorted to indirect methods, such as those that Hodgkin and Huxley had applied in their classic papers, i.e., if they could not directly measure a desired parameter, they would devise a protocol to measure it indirectly. The results they recorded with high-frequency firing suggested an accumulation of K^+^ immediately outside of the squid axon [18]. Hodgkin and Katz demonstrated that the squid giant axon was not exclusively permeable to K^+^ at rest, thus they could not directly apply the Nernst equation to estimate changes in membrane potential in response to estimated changes in [K^+^]_o_, assuming that [K^+^]_i_ remained unchanged [9]. Instead, they simply correlated known concentrations of K^+^ in the seawater bathing the axon with changes in membrane potential in order to calibrate experimental measures of the membrane potential to assumed increases in [K^+^]_o_. This calibration established a near linear relationship of the membrane potential versus [K^+^]_o_ plotted on a log scale. The data implied that even after modest action potential firing, significant extracellular K^+^ accumulated, i.e., greater than 1 mM for a single action potential, which could reasonably be explained by a semi-permeable membrane tightly surrounding the axon, forming a reservoir whose small volume concentrated the exuded K^+^. The diffusion of K^+^ from this reservoir into the interstitial space could be estimated based on the time taken for the action potential to return to its baseline profile. These data were of fundamental importance to brain function as they demonstrated the previously unexpected consequence of action potential conduction affecting the waveform of subsequent action potentials. Given the reliance of brain function on frequency encoding, this was clearly a topic worthy of further study.

## 4. Selective K^+^ Permeability of Glial Cells

**Figure 2:**
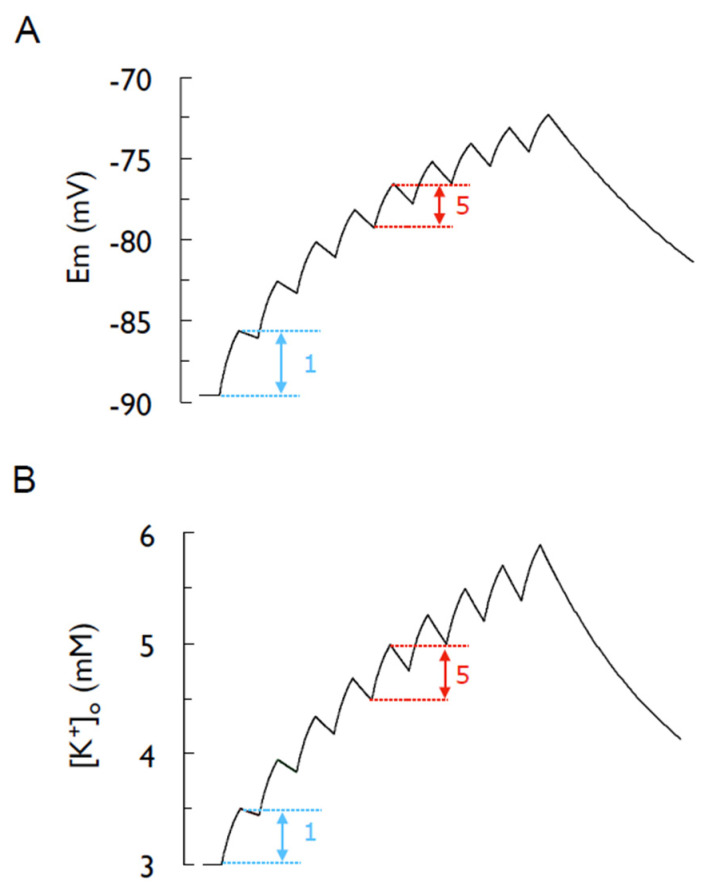
Stimulus-induced depolarisation of the glial cell membrane potential in *Necturus* optic nerve. (**A**) A model based on Em = 58 log ([K^+^]_o_/99), illustrating the glial cell membrane potential depolarization in response to 9 sequential stimuli at 8 Hz, where each stimulus liberates the same amount of K^+^. (**B**) The elevation on [K^+^]_o_ that produces the depolarization in (**A**). Note how each stimulus raises [K^+^]_o_ by the same amount. Note how the arrows in (**B**) denoting the rise in [K^+^]_o_ associated with the 1st and 5th stimuli are identical in amplitude, but the membrane depolarisation associated with the 1st stimulus is larger than that for the 5th.

The next important study relating to activity-induced changes in extracellular K^+^ was carried out in the amphibian mudpuppy *Necturus* [22,23]. The introduction of glass microelectrodes in 1949 [24] was a major technical breakthrough in the field of neuroscience as it allowed researchers to move away from large invertebrate axons to preparations more related to mammals. This started with studies on large cells, such as the R15-identified neurones in *Aplysia* [25], and ultimately graduated to recordings from single mammalian neural cells. Stephen Kuffler was one of the first researchers to use this technique to investigate activity-induced increases in [K^+^]_o_. The mudpuppy optic nerve offered the advantages of being amenable to in vivo and ex vivo intracellular recordings. Kuffler et al. identified a role for glial cells in the mudpuppy, equivalent cells to astrocytes in mammals. This was the first physiological function ascribed to glial cells and would ultimately have universal significance in introducing the concept of neuronal glia interactions, where the behaviour of one cell type influences the behaviour of the other [26]. Kuffler impaled cells in the *Necturus* optic nerve with the microelectrode and recorded a very negative membrane potential, identifying the cell as a glial cell. When the optic nerve was stimulated, action potentials would be conducted along the length of the axons, bypassing the glial cells, which were in close association to the axons. The glial cells responded to action potentials with a membrane depolarisation, suggesting they were responding to the K^+^ released from the active axons. This effect was quantified by altering the [K^+^] in the artificial cerebrospinal fluid (aCSF) bathing the nerve and measuring the membrane potential response. The relationship was linear when [K^+^] was plotted on a log scale, indicating that the glial cells responded in a manner predicted by the Nernst equation to changes in [K^+^], suggesting they could be considered as K^+^ electrodes. Thus, the reversal potential (E_K_) changed from being a concept to a potential that could be measured experimentally. Bearing in mind the Nernst equation contains three parameters, the value of [K^+^]_o_ could be estimated from the measures of the membrane potential if [K^+^]_i_ was assumed to remain unchanged [27]. The stimulus of the axon produced a slight depolarization of the glial cell membrane and straightforward calculations using a rearrangement of the Nernst equation, where [K^+^]_o_ was the unknown parameter, estimating the magnitude of [K^+^]_o_ that would cause a depolarisation of known magnitude. Furthermore, based on the Hodgkin and Huxley data, it was assumed that every action potential released an identical amount of K^+^. This limited information could be used to reveal considerable information regarding glial cell behaviour compared to neurons. (1) The glial cell membrane potential is more hyperpolarised than the neuronal membrane and is estimated from the Nernst equation, (2) for a given increase in extracellular K^+^, the glial cell membrane potential will depolarise to a greater extent than the neuronal, (3) Kuffler went further and proposed that glial cells buffer the extracellular K^+^, absorbing it and distributing it throughout the glial cell network, then releasing it back into the extracellular space when the concentration of extracellular K^+^ had fallen. This is a topic we shall return to later. (4) Sequential action potentials produced the depolarisation of the glial cell membrane of decreasing amplitude (Figure 2), as expected from the logarithmic relationship between [K^+^]_o_ and membrane potential, (5) and when the membrane potential reaches 0 mV, [K^+^]_o_ must equal [K^+^]_i_ and this occurred when [K^+^]_o_ was increased to 99 mM; thus, [K^+^]_i_ was assumed to equal 99 mM. This demonstrated the utility using the Nernst equation as a tool in devising experimental design [28], the power of such an approach obvious when one considers estimates of [K^+^]_i_ could be accurately made many years before [K^+^]_i_ could be directly measured experimentally.

## 5. Pseudo-Nernstian Response of Mammalian Astrocytes to K^+^

In the early 1970s, Bruce Ransom, working with Sydney Goldring, demonstrated in the living cat cortex that astrocyte membrane potential was sensitive to changes in extracellular K^+^, although the response was not Nernstian as expected, i.e., a change of about 60 mV for an order of magnitude change in [K^+^] bathing the cell [29]. Instead, the response was only about 40 mV, which at the time was viewed as a disappointment, but in hindsight was an important observation as it indirectly shed light on two important physiological processes that contribute to the regulation of interstitial K^+^, tortuosity of the interstitial space [30] and astrocyte buffering [31]. The electrode penetrated the tissue up to 400 µm below the pial surface with the reservoir of aCSF on the surface of the cortex containing K^+^ of various concentrations. When the solution was changed, the relatively large distance from reservoir to electrode meant that K^+^ did not reach equilibrium, since the tortuosity of the ECS ensured the diffusion of K^+^ though the interstitial space was hampered. In addition, the K^+^ was buffered by astrocytes, hence the K^+^ surrounding the astrocyte penetrated by the electrode was less than that in the surface reservoir, which would lead to E_K_ being smaller than estimated [32].

Interstitial ions concentrations are rigorously maintained within a narrow range to ensure an optimal environment exists for neuronal function. However, ions present in the interstitial space exist in dynamic equilibrium with intracellular compartments [33]. Changes in interstitial ion concentrations may be localised to distinct regions, e.g., as a result of synaptic activity, or may be more general as occurs in nerve tracts, such as the acutely isolated *in vitro* optic nerve preparation, where supra maximal stimulus excites all available axons, leading to a uniform elevation in [K^+^] throughout the interstitial space [34,35]. The advent of ISMs (see below) in the 1960s ensured a vast literature accumulated relating to Ca^2+^ [36] and H^+^ [37], but predominantly to K^+^ [38,39,40], since it is the sole determinant of astrocytic membrane potential [41] and the predominant determinant of neuronal membrane potential [13]. Interstitial [K^+^] directly affects the membrane potential of neurones and astrocytes in a predictable manner [27,42], which, in turn, affects neuronal excitability and synaptic transmission. These studies established baseline values of interstitial ions in vitro and focused on activity-related changes in interstitial [K^+^], explained as the epi-phenomenon of neuronal activity, a reasonable assumption given the rapid return of [K^+^]_o_ to baseline values on the cessation of activity [34,35]. Although such studies were focused on neuronal activity, they also informed on the buffering capability of astrocytes, which limited the increase in activity-dependent [K^+^] to about 12 mM [34], the physiological ceiling level, maintained by Na^+^-K^+^-ATPase and Kir channels [34,35] on astrocyte membranes, which act as sinks for the intracellular astrocytic accumulation of K^+^, followed by the dispersion of K^+^ via spatial buffering [43], where electrotonic repulsion releases K^+^ into interstitial space distant from the site of activity.

Interest in the measurement of interstitial [K^+^] by ISMs waned in the wake of the development of new technologies in the 1980s, such as molecular biology, immunohistochemistry, MRI, biosensors and latterly optogenetics, lending the associated literature a historic feel. However, more recent data has suggested alterations in interstitial ions drive physiological activity rather than result from it, with K^+^ playing a leading role [44]. It is proposed that interstitial ions regulate the transition between the brain states, sleep and wakefulness [45]. Such developments are, in part, due to technical advances in the in vivo monitoring of interstitial ions, such as K^+^, Ca^2+^ and Mg^2+^, coupled with the simultaneous EEG recording of brain activity, allowing for correlation between brain state and interstitial ions [45]. The distinction between sleep and wakefulness can be accurately assessed using EEG [46]; the former is characterised by synchronous neuronal activity and 0.5 to 1 Hz delta oscillations, whereas wakefulness is characterised by desynchronised neuronal activity and suppressed delta oscillations. Real time measures show rapid [K^+^]_o_ increases of 0.4 mM, whereas Ca^2+^ and Mg^2+^ decreased by about 0.1 mM on the transition from sleep to waking [45]. Such changes in interstitial ions could be considered epi-phenomena; thus, the authors addressed this vital issue of whether imposing interstitial ion concentrations associated with identifiable brain states would alter behaviour, which was convincing evidence of a role for interstitial ions in determining behaviour, rather than resulting from it. Infusing aCSF-containing ion concentrations associated with wakefulness in asleep mice decreased EEG activity and delta oscillations, indicating an awake phenotype, which was reversed when infusion ceased. Infusing ‘sleep’ aCSF produced a reduction in EMG activity, which was revered when infusion stopped [45].

The alterations in ion concentrations would appear too small to have such a large impact on activity [13]. However, they likely act in concert, suggesting steep voltage dependence of affected channels, such as the low-threshold T-type Ca^2+^ channels, and an exquisite sensitivity of Ca^2+^-dependent K^+^ channels, as well as the inhibition of the membrane stability imposed on membranes by divalent cations, such as Ca^2+^ and Mg^2+^ [47]. Reduced Mg^2+^ would also increase NMDA receptor activity [48]. The change in interstitial ions is likely due to neurotransmitter release associated with sleep, such as acetylcholine, orexin, serotonin, dopamine and histamine. The experiential application of these compounds increased interstitial [K^+^] in the presence of TTX [13], demonstrating the effect was not due to neuronal activity. Thus, it appears that neurotransmitters can affect EMG via a direct established effect on neurones and a novel indirect effect via the modulation of interstitial ion concentrations.

## 6. Ion-Sensitive Microelectrodes

**Figure 3:**
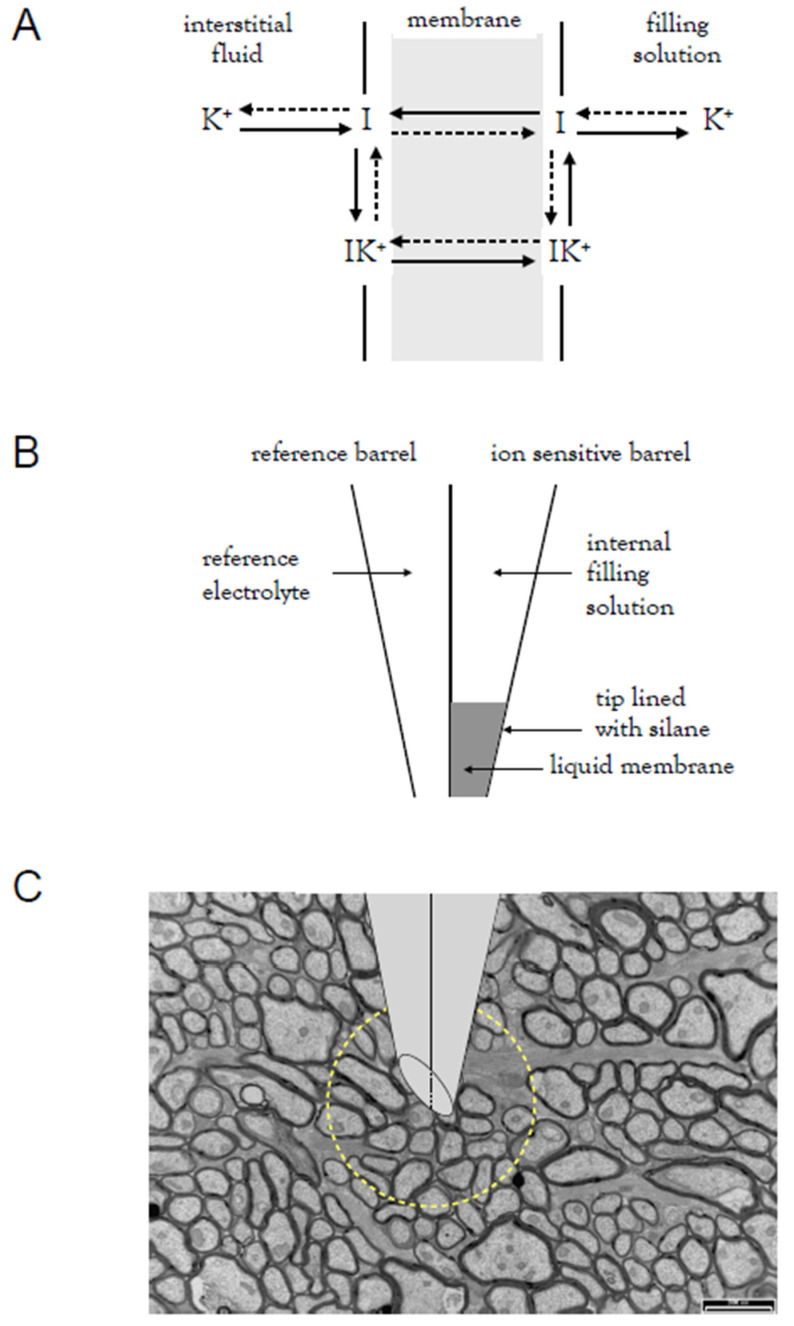
Ion-sensitive microelectrodes. (**A**) The liquid membrane is highly selective for K^+^ and extracts the ion from bulk solution, in the case of an experiment, from the interstitial fluid and by carrier translocation deposits the K^+^ to the internal filling solution compartment. (**B**) Diagram of double-barrelled ISM showing the reference barrel on the left filled with reference electrolyte, and the resin-filled barrel on the right. The silanised tip retains the hydrophobic resin. (**C**) Electron micrograph of adult mouse optic nerve shown in transverse section. The scale bar is 2 μm. All axons in the adult animal are myelinated. The appropriate dimensions of the tip of an ISM have been superimposed. The yellow circle encloses an assumed volume of interstitial fluid adjacent to the tip of the ISM.

The application of the Nernst equation to estimate concentrations of extracellular ions relies on a series of assumptions [27] that necessarily introduce a degree of error to the results [18]. The advent of ion-sensitive membranes as applied to ion-sensitive microelectrodes [49] allowed accurate, real time measures of extracellular ions, which could be temporally correlated with imposed stimulus [50]. For the purposes of this paper, we will focus on K^+^, but the technique has been applied to other common physiologically relevant cations, such as Ca^2+^ [51] and H^+^ [52]. The theory underlying ISM involves the use of a membrane to separate a solution containing an ion of interest (Figure 3B); in this review, this is the interstitial space and a reference solution of known ionic composition [53]. The membrane is selectively permeable to the ion of interest [54], which generates a voltage when it interacts with the membrane [55]. There are three types of membrane: glass, which has been used to measure pH; crystalline membrane made of inorganic insoluble salts; and wet liquid membranes, which will be the focus of this review. Liquid membranes have been devised that are exclusively sensitive to a particular ion and comprise the following components: a hydrophobic solvent, a neutral lipophilic ionophore that complexes with K^+^, and hydrophobic counterions [49]. The liquid membrane occupies a plane between two aqueous solutions; in the case of ion-sensitive microelectrodes (ISM), the interstitial or extracellular fluid and the electrode solution. The ionophores for K^+^ are either antibiotics, such as valinomycin, or cyclic ethers. The nature of valinomycin promotes binding to K^+^ present in the interstitial space but to no other ions. The K^+^ is then transported across the plane and released into the electrode solution, thereby creating a flow of current (Figure 3A).

**Figure 4:**
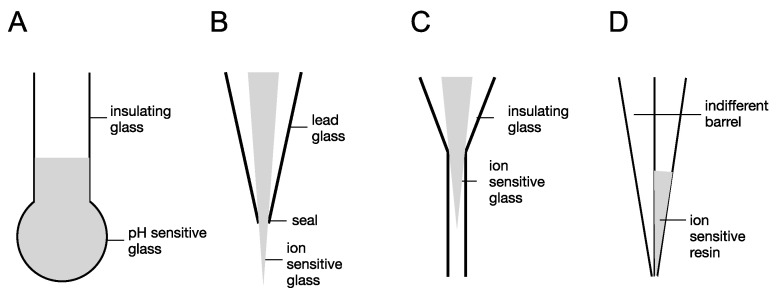
Evolution of ISMs. (**A**) H^+^-sensitive glass was used to sense pH changes and was the foundation of laboratory pH meters. (**B**) Ion-sensitive glass for microelectrodes that sensed Na^+^ and K^+^ had long protruding tips limiting their use to large cells. (**C**) Recessed tip glass microelectrodes increased the range of cells that could be studied. (**D**) Double barrelled ISMs that used liquid ionophores allowed recording from smaller mammalian cells and increased the number of ions that could be studied.

The evolution of ISMs may be traced to the discovery of glass sensitive to H^+^ that was used to measure pH. This technology was later miniaturised to allow the measurement of intercellular pH [56] from cells of a sufficiently large size (Figure 4A). From this, other glasses were developed that were sensitive to other physiologically relevant cations, such as Na^+^ and K^+^ [57]. These electrodes consisted of the inner ion-sensitive glass filament shaped to a long (100 µm) narrow tip [58], enclosed in an outer layer of lead glass to provide physical insulation from the sample (Figure 4B). This profile had the disadvantage of a relatively long section of ion-sensitive glass, which required a large sample of tissue, or large cells, if intracellular concentrations were being studied. The issue was remedied with the development of tips only a few µm in length [59]. A recessed tip configuration was produced in which the ion-sensitive tip was enclosed with the shielding layer of outer glass (Figure 4C), which allowed smaller cells to be studied, since only the tip had to enter the cell. However, the use of liquid membranes [60] was an enormous step forward as it expanded the range of physiologically relevant ions that could be studied, and the small tips of such electrodes (Figure 4D) allowed recordings from small mammalian cells with much more clinical relevance that the large non-mammalian cells, such as *Helix* gastropod models [61].

These ISMs have limitations, including that their invasive nature damages tissue on the insertion and placement of the electrode (Figure 3C) and that they are limited to single point measurements [62]. They generally record from a large pool of interstitial fluid at the electrode tip, which may affect their sensitivity, since the pool will dampen fluctuations in ion concentrations. However, they do provide real time measurements of ion activities, which renders them very useful when compared to the dialysis technique, a far more invasive and difficult technique to implement, which offers only post hoc estimates of ion concentrations at a single time point, limiting their use [63].

Whatever the ion of interest, ionophores ideally share the following key features:

1. Selectivity and specificity. The ionophore is specific to only one ion and is sensitive to changing concentrations of the ion. Important considerations for the measurements of K^+^ are that the ionophore is insensitive to Na^+^, which occurs in the interstitial space at concentrations significantly greater than K^+^ and are unaffected by pH, where metabolic experiments can alter the [H^+^] by up to an order of magnitude [51,52].
2. Sensitivity. The ionophore can record low concentrations of the ion.
3. Real time measurement. Allows correlation with simultaneously recorded electrical activity, such as evoked activity in in vitro preparations or EEG recordings in in vivo behaving mice.
4. Small size. Can record from localized identifiable brain regions, e.g., hippocampal structures [64], layers of retina [65], etc.
5. Low cost to produce and relatively easy to manufacture [66,67].

The basic design of ISMs is constantly being optimised. The development of solid contact ISMs in which the liquid reference solution in the barrel is replaced by a solid contact material simplifies manufacture, removes the need for calibration and prolongs the life of the electrode. The solid compound is based on conduction polymers, of which there are a variety of carbon-based materials, such as graphene. Although beyond the scope of this review, more detailed descriptions of the development, properties and applications of solid contact ISMs are available [68,69,70]. An additional refinement applied to solid contact ISMs is to coat the ion-sensitive tip with a permeable membrane of silicone rubber that does not interfere with the sensing capability of the ionophore, but prevents the build-up of biological material (biofouling) on the electrode and significantly prolongs the lifetime of the electrode whilst maintaining its sensitivity [71,72].

The properties of ISMs described above limit the applications of the electrodes, particularly in vivo. An emerging technology is optogenetics in which light is used to produce biological effects. Channel rhodopsins have been used to depolarise cells by directly activating ion channels, but a recent study has shown that this technique results in rapid transient elevations in interstitial [K^+^] up to 5 mM, so care must be taken in interpreting data derived from this technique [73]. In addition, there are promising advances in the use of carbon nanotubes as electrochemical sensors, with measurements of Ca^2+^ being demonstrated [74]. An alternate means of measuring [K^+^] is the development of K^+^-sensitive nanosensors. These sensors were created by trapping a commercially available K^+^ indicator inside the core of mesoporous silica nanoparticles (MSNs). The excitation of the particles with near ultra violet light excites the K^+^ indicator via luminescence resonance energy transfer. The nanosensor is covered with a thin layer of a K^+^-selective membrane, into which micropores created from carbonyl oxygen based on the structure of K^+^ channels are incorporated, ensuring selectivity for K^+^. Imaging techniques are used to estimate the K^+^ concentration as a fluorescent signal that is linearly related to [K^+^] [75]. The nanosensors can be recycled after use. The utility of these nanoparticles has been demonstrated in single cells, brain slices and live mice. In cultured neurones, the nanoparticles allow for spatiotemporal resolution of K^+^ release, an important advance in studies on spatial buffering. In brain slices, the nanoparticles allow a simultaneous measurement of [K^+^] in several regions, allowing the visualisation of the spatiotemporal changes in [K^+^] in response to physiological stimulus. Most impressively, the nanoparticles were injected into the mouse hippocampal CA3 region and the response of [K^+^] to seizures of increasing intensity was recorded. The nanosensors did not evoke any immune response nor did they affect neuronal excitability or the resting membrane potential of neurones. As expected, the [K^+^] during grand mal seizures increased towards 10 mM [76], results similar to those recorded with ISMs [39]. The advantages of this new technology are the spatiotemporal resolution, the non-invasive nature of the sensors and the ability to measure [K^+^] in freely moving mice. Refinements in the technique may allow for the mapping of the epileptic focus and may be potentially engineered as a drug delivery mechanism providing anti-epileptic drugs at the origin of the seizure.

The range of techniques for measuring ions each comes with its own pros and cons. For the ISM, the advantages include that they allow the continuous measurement of ion activity in real time; they omit measurement from intracellular organelles; more than one ion can be simultaneously measured; they are relatively easy to make and the cost of associated equipment is low; and calibration is straightforward, relying on a Nernstian relationship between the output voltage and the estimated [ion]. The disadvantages include that intracellular measurements are not possible from small cells, e.g., cerebellar granule cells, microglia, etc.; the specificity may be limiting; they are prone to electrical interference requiring the use of a Faraday cage; they require dexterity on the part of the experimenter for the accurate placement of ISM; the response time is not instant; and ISMs can only record from one location [77]. Dialysis has the advantage of sampling all ions in the interstitial fluid and allowing recovery experiments in vivo, which permits longitudinal studies where the effects of an intervention can be followed up for periods of days or even weeks. The disadvantages include the tissue damage incurred by the placing of dialysis probes; the post hoc nature of the estimates of ion concentrations, i.e., not in real time; the sampling of the interstitial fluid only provides an average concentration in a large volume; and they can only record from one location [78]. The imaging technologies where fluorescent signals are recorded, such as with nano-sensors, have the advantage that they can record from more than one location, they can record longitudinally over time, they can be used to record in vivo, they have a very rapid response time, they allow for continuous measurement and they do not require manipulative skill. However, the disadvantages include that the technology is costly and limited to those with skill in fluorescent imaging; the bleaching of dyes, photo-toxicity and indicators are not yet available for all physiologically important ions; and calibration is not straightforward [77].

There are numerous variations in the construction of ISMs but most follow this pattern [49,79]. Double-barrelled glass micropipettes are pulled to a fine tip. One tip will contain the ion-sensitive resin, the other is a reference electrode for the subtraction of ambient electrical interference, such as activity-dependent direct current (DC) shifts. The ion-sensitive barrel is back filled with a small volume of silane. The electrode is then heated to 160 °C for 60 min to evaporate the liquid silane and create a hydrophobic coat lining the lumen of the electrode tip. The electrode is then bevelled or broken back to a diameter of between 1 and 5 µm to ensure a sufficient area of resin is present for the absorption of K^+^ to produce significant current flow. The electrode is back filled with a solution resembling the osmolarity and pH of the interstitial fluid. The tip is then front filled with the liquid membrane, whose hydrophobic nature bonds with the silanised tip. The goal of using ISMs is to record in real time the activity (concentration) of an ion of interest, in our case K^+^, but the ion-sensitive barrel will also record local electrical activity resulting from changes in the membrane potentials of cells abutting the electrode tip. To mitigate this unwanted electrical interference, these local potentials, also called DC shifts, are recorded via a separate reference barrel. The tips of the ion-sensitive and reference barrels must be isopotential; thus, double-barrelled glass is the preferred method. The reference barrel is filled with an appropriate solution to minimize junction potentials; in the case of K^+^ it is 140 mM KCl, 20 mM (4-(2-hydroxyethyl)-1-piperazineethanesulfonic acid) (HEPES) buffered to pH 7.2. The reference barrel signal is subtracted from the ion-sensitive barrel by a differential amplifier, isolating the potential due to the ion activity of interest [49,80]. An IV converter in the recording amplifier converts the K^+^ current flow into a voltage, allowing estimates of [K^+^]_o_ by applying the Nernst equation. For detailed descriptions of fabrication and implementation of ISMs, consult the following references [49,62,79].

There are several issues that must be borne in mind when using ISMs. The first is that ISMs are sensitive to vibration, so use of a vibration-isolating air table is essential, and motorised micromanipulators are recommended, but not essential, for the placement of the ISM in the tissue. The second is that electrodes must be calibrated before and after use to ensure the fidelity of the signal recorded. Nernstian responses are expected of ISMs with orders of magnitude deviations of ideally over 50 mV for monovalent cations. Thirdly, and most importantly, the dimensions of the tip of the electrode relative to the cellular components present in the tissue is an important consideration in data interpretation. Although the electrode is considered a ‘micro’ electrode, it is still large compared to the size of individual cells. The purpose of bevelling the tip of the electrode is to create a profile that easily penetrates the tissue in the manner of a hypodermic needle. In addition, the relatively large size of the tip ensures it will not penetrate cells, so there is no scope for intracellular recording, which would confuse data interpretation. The forward movement of the electrode into the tissue destroys cells and releases their intracellular contents into the interstitium. This has implications, particularly for recordings of K^+^ and Ca^2+^, where the interstitial concentration of these ions varies by orders of magnitude from their intracellular concentration. The result of ISM penetration would be a dilution of interstitial Ca^2+^ but a concentrating of interstitial K^+^. Thus, once the ISM has been inserted into the tissue, at least 30 min is required for homeostatic equilibration to restore the ion to its baseline concentration.

## 7. Calibration of ISMs

**Figure 5:**
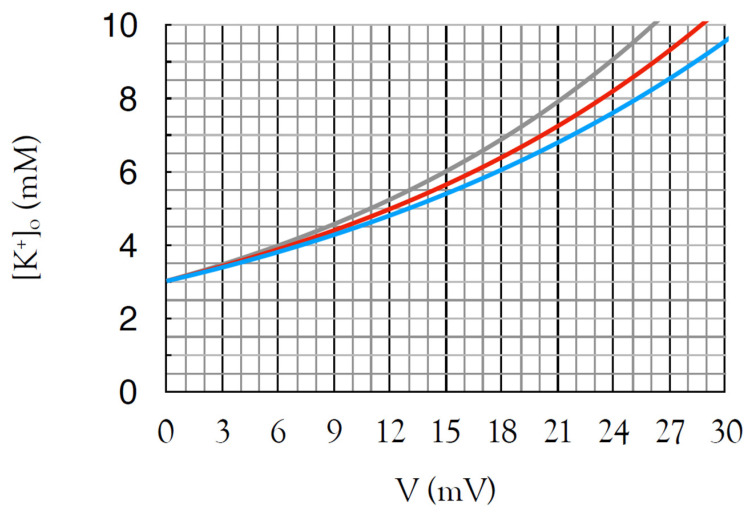
Calibration curve for ISM according to rearrangement of the Nernst equation with a bathing aCSF of 3 mM K^+^. Curves are for electrodes calibrated in 3 mM and 30 mM K^+^ with responses of 60 mV (blue), 55 mV (red) and 50 mV (grey), which give voltage deflections of 18 mV, 16.5 mV and 15 mV, respectively, for increases in [K^+^]_o_ from 3 to 6 mM.

When choosing calibration solutions, care should be taken to use appropriate concentrations, which requires a firm understanding of the implications of the Nernst equation [27]. For example, the interstitial K^+^ in ex vivo tissue, such as the optic nerve model that we describe, is approximately 3 mM when superfused with 3 mM KCl. Under physiological conditions, interstitial K^+^ in the adult rodent optic nerve does not exceed 10 mM [50]. It is desirable to maximise the signal to noise, and an appropriate calibration range would be 3 mM, 9 mM and 30 mM K^+^ (Figure 5). The amplifier voltage is adjusted to 0 mV in 3 mM K^+^ and should approach 60 mV in 30 mM K^+^ (for the purposes of illustration we will assume it is 55 mV). The ISM is then inserted into the nerve, and once the signal has equilibrated, denoted by a stable, unchanging signal, the amplifier voltage is manually adjusted to 0 mV. If an experimental manoeuvre is carried out that increases K^+^ to 6 mM, this will register as an increase of 16.5 mV (55 mV × 0.301: log_10_2 = 0.301) on the amplifier, a sufficiently large signal that may only require x10 amplification prior to computer acquisition.

The following must also be kept in mind. As a result of neuronal activity, a general assumption is that the K^+^ moves directly from the neuron/axon into the interstitial space. However, the sub-cellular structure of axons and the interstitial space must be considered when assessing data. As mentioned above, the tip of the electrode is larger than the cell bodies, and thus damages the cells bodies, releasing ions into the interstitial space. In a cylindrical tissue, such as the optic nerve, where action potential propagation in response to supra-maximal stimulus is longitudinally down the tract, the electrode can only record from a very limited volume of tissue but is assumed to represent what occurs in all regions of the nerve. This may not be the case. Although the interstitial space occupies a relatively large volume of this tissue (about 15%) [31], due to membranous invaginations, the distance between cells is very narrow (fractions of a micron). The tortuosity of the interstitium hinders the flow of ions, leading to localized ion accumulation, which does not rapidly diffuse to neighbouring regions. It would be interesting to assess the rise of [K^+^]_o_ in the mouse optic nerve in response to a standard supra-maximal stimulus where the K^+^-sensitive microelectrode is sequentially inserted into different regions of the nerve.

Another consideration is how the structure of myelin, which ensheathes axons in white matter tracts, affects K^+^ movement. The conventional view of the location of ion channels at the node of Ranvier places Na^+^ channels at a high density in the node, along with delayed rectifier K^+^ channels. However, immunohistochemical studies have localized K^+^ channels to the axonal membrane in the juxta-paranodal region [32]. These channels are not considered to contribute to the repolarizing phase of the action potential, which is overseen by high conductance leak channels present at the nodes. The recordings of interstitial K^+^ support this for the following reasons [33]. The tight junctions that insulate the sub-myelin space from the interstitium [34] would limit K^+^ movements, inconsistent with the rapid increase in [K^+^]_o_ in response to stimulus. Ion movements through leak channels (predominantly K^+^) respond in an Ohmic manner to membrane potential, and thus the large depolarization at the peak of the action potential, in conjunction with the rapid inactivation of nodal Na^+^ channels, would produce a large voltage gradient that would generate a leak current sufficient to rapidly repolarize the membrane potential.

The location of nodes on axons of different diameters must also be considered. It is accepted that the inter-nodal length is a fixed ratio relative to axon diameter [35]. Given the variable diameters of axons in tracts, such as the optic nerve, the nodes of Ranvier of neighbouring axons will not align along the length of the nerve, such that a brief period of activity that results in a small K^+^ release from the node of one axon is unlikely to affect the membrane potential of neighbouring axons, whose nodes are sufficiently distant that the K^+^ will be buffered before it can diffuse any significant distance. Thus, one must be mindful in generalizing the effects of one individual axon to a population of axons.

## 8. Physiological and Pathological Limits for [K^+^]_o_

Although axons release K^+^ to the interstitial space and axons are found in both grey and white matter, there are considerable differences between the two tissues. In white matter, myelinated axons run in parallel tracts allowing simultaneous stimulation of all axons within the tract [81], leading to the coordinated release of potassium [50]. Under physiological conditions, e.g., in the eye, axon activation is dependent on stimulus and not all axons are activated simultaneously; thus, physiological implications derived from experimental data must be interpreted with caution. However, in grey matter during pathological conditions, such as epilepsy, synchronised high-frequency firing of neurons occurs over a considerable volume of the brain, leading to localised elevations in extracellular K^+^ [82].

**Figure 6:**
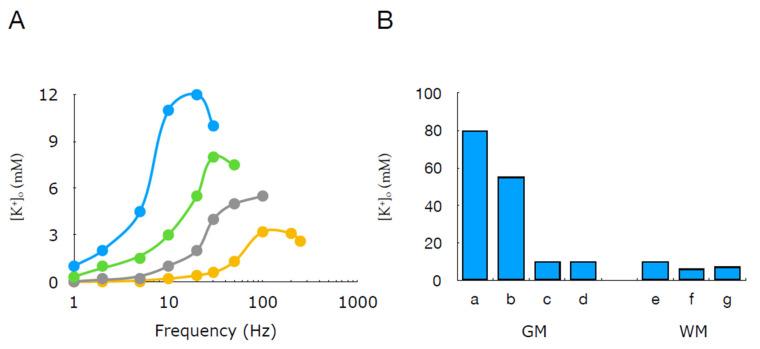
Elevations in [K^+^]_o_ in grey and white matter. (**A**) In rat optic nerve, the frequency dependent elevations in [K^+^]_o_ are age-dependent, with the largest increases occurring in the youngest rats (blue = 2 days, green = 6 days, grey = 13 days, orange = adult). (**B**) Increases in [K^+^]_o_ in grey (GM; a–d) or white matter (WM; e–g) associated with a = anoxia, b = spreading depression, c = seizure, d = 100 stimulus, e = anoxia, f = aglycaemia or g = 100 Hz stimulus (adapted from Ref. [96]).

The limits to which interstitial K^+^ can rise will be discussed first in terms of white matter, since this topic has been extensively studied, and the relatively simple structure of white matter tracts simplifies interpretations of the results. The rodent optic nerve has been the model of choice [50], with the most significant contribution coming from Bruce Ransom’s lab. The rodent optic nerve is an ideal preparation as it can be dissected easily with minimal damage to the tissue [81]. It lacks the complications of glutamatergic synapses and consists of a simple morphology, with myelinated axons running in parallel interspersed with oligodendrocytes and astrocytes [83,84,85]. The cylindrical nature of the tissue means it is ideal for electrical stimulation with suction electrodes [86] and is amenable to penetration with ISMs as the diameter of the rat optic nerve is up to 600 μm [87]. When the nerve is stimulated at high frequency with a train of action potentials, a rapid elevation in [K^+^]_o_ is seen, which rapidly returns towards baseline on the cessation of stimulus [34,50,88]. The optic nerve from a neonatal rat is translucent in appearance, indicating its lack of myelin and its immature nature [89]. The stimulus-evoked compound action potential has a bipolar profile, which transforms into the adult triple peaked profile on reaching maturity [90] and is considered to occur at 45 days [91]. In 1- to 3-day-old rat optic nerves, a 10 s train of action potentials at 20 Hertz (Hz) causes [K^+^]_o_ to rise to 17.2 mM from a baseline of 5 mM (Figure 6A). Increasing maturity leads to the attenuation of the K^+^ increases with comparable stimulus, leading to a K^+^ rise to 9.8 mM in the adult [50]. These data indirectly suggest that glial cells, which become abundant upon maturity, play a role in buffering elevations in interstitial K^+^ reflected in the lowered ceiling level. This is supported by data in which gliogenesis was interrupted by 5-azacytidine (5-AZ), where the K^+^ ceiling upon stimulus was increased in treated rats compared to untreated animals [92].

In response to metabolic insults, [K^+^]_o_ also increases, the mechanism presumably related to the decreased supply of ATP leading to Na^+^-K^+^-ATPase dysfunction and compromised buffering. In adult rat optic nerves, imposing anoxia by replacing O_2_ with N_2_ caused [K^+^]_o_ to rise to 14 mM from a baseline level of 3 mM [93]. Interestingly, the degree to which [K^+^]_o_ rose during anoxia was related to the concentration of ambient glucose, with lower glucose leading to greater elevations in [K^+^]_o_: in 5 mM glucose, [K^+^]_o_ exceeded 15 mM but only reached 7 mM in 20 mM glucose. When glucose was removed (simulated aglycaemia), the [K^+^]_o_ slowly rose from a baseline of 3 mM to 6 mM after 60 min of insult [51].

The diversity of grey matter structures in the brain, e.g., hippocampus, brain stem, cerebellar layers, etc., precludes the concept of representative regions in the way that optic nerve, corpus callosum or ventral tracts of the spinal cord can be considered representative of central white matter. Most data relevant to this review derives from experiments on rodent cortex, which qualitatively agree with white matter studies, where high-frequency stimulus leads to elevations in [K^+^]_o_, although cortical values tend to be larger in amplitude. Experiments in cortex revealed that stimulus produced [K^+^]_o_ elevations up to 10.2 mM from a baseline of 3 mM at frequencies of 20 to 25 Hz [38]. Anoxia produces rapid and very large increases in [K^+^]_o_, approaching 100 mM. Such large increases suggest uncontrolled K^+^ release and interstitial accumulation, where [K^+^]_o_ approaches the value of [K^+^]_i_ [94]. Grey matter is susceptible to pathologies not associated with white matter. The spreading depression of Leão is an exclusively grey matter phenomenon [95]. It was initially demonstrated experimentally in rodent brain and involves a slow depolarising wave that spreads across the cortex at about 3 mm min^−1^, which inhibits all electrical activity in its wake. There follows a refractory period and then the restoration of normal activity [96]. The phenomenon is thought to occur in humans and may be associated with the scotoma of migraine [97]. Measurements in experimental animals have shown increases of up to 55 mM [98]. During epilepsy, synchronised neuronal discharges lead to elevations in [K^+^]_o_ that can exceed 10 mM [39], but whether the elevated [K^+^]_o_ is a cause or effect of the seizure is a widely debated issue [82]. A picture thus emerges of neural activity leading to elevations in [K^+^]_o_ (Figure 6B). The studies by Kuffler precipitated the assigning of a role for glial cells (astrocytes) in re-equilibrating [K^+^]_o_, probably the first example of neuronal glia interactions [86] and the first physiological function ascribed to astrocytes.

## 9. Interstitial K^+^ and Lactate

The metabolic inter-dependency of astrocytes and neurones most likely evolved to relieve the metabolic burden of neuronal signalling solely from neurones, such that astrocytes actively contribute to supplying energy substrate to active neurones. The intermediary molecule that sends signals to the astrocytes of increased energy requirements of active neurones was initially proposed as glutamate [99] but has since been superseded by interstitial K^+^ [100,101]. This makes sense since K^+^ is released from all active axons and, therefore, can be regarded as a universal signalling molecule [102]. When considering a suitable signalling molecule, the following criteria must be met: (1) the concentration of the molecule must be in direct proportion to the neuronal metabolic demands, (2) the astrocyte must be sensitive to the molecule, and (3) there must exist a mechanism of clearing the signalling molecule on the cessation of neural activity. Interstitial K^+^ meets these criteria. Studies in grey matter have indicated that elevations in interstitial K^+^ activate a Na^+^-coupled HCO_3_ transporter present on the astrocyte cell membrane that leads to intercellular alkalisation in astrocytes. This activates soluble adenyl cyclase to produce cAMP, which causes glycogen metabolism and the release of lactate as a conduit that is taken up by neurones for oxidative metabolism [103].

## 10. Interstitial K^+^ Buffering

**Figure 7:**
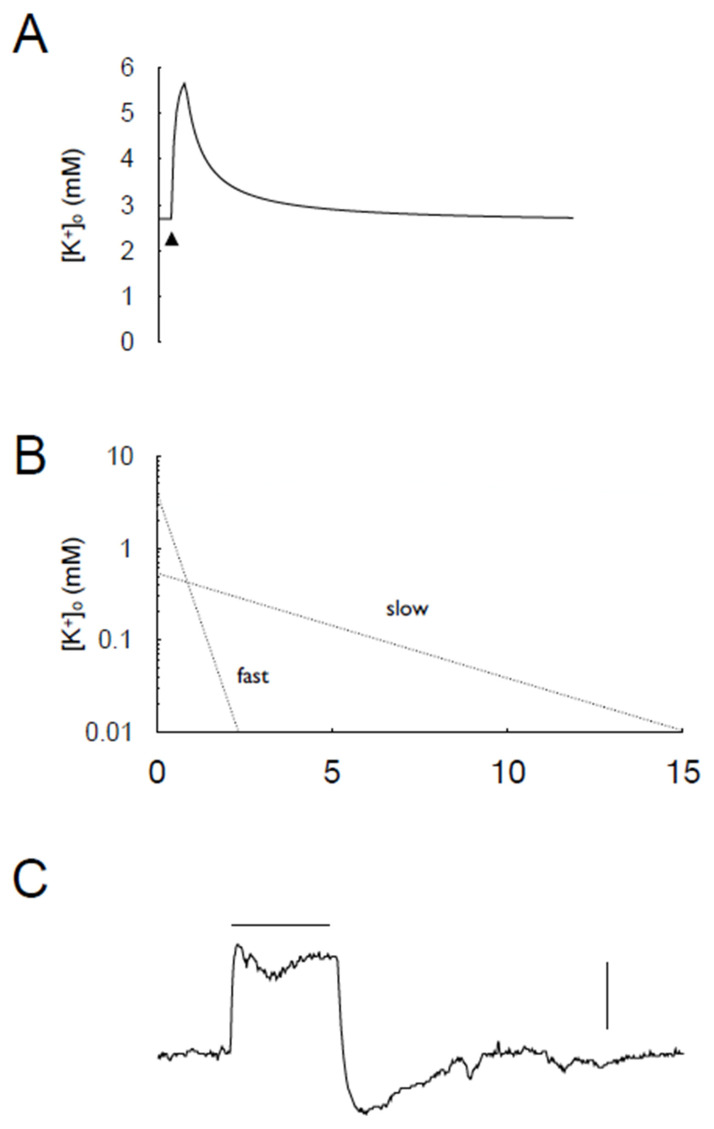
Buffering of interstitial K^+^ in rodent optic nerve. (**A**) A stimulus of 100 Hz for 10 s leads to a rapid increase in [K^+^]_o_, which declines rapidly on cessation of firing. (**B**) The buffering of K^+^ can be ascribed to two processes, characterised by a fast or slow time constants (adapted from Ref. [34]). (**C**) Imposing 100 Hz stimulus for 2 min (horizontal bar) in mouse optic nerve leads to a transient increase in [K^+^]_o_ (scale bar = 1 mM) that plateaus despite continuing stimulus, followed by a rapid fall in [K^+^]_o_ below baseline before recovering to baseline concentration.

Optimal brain function requires the rigid control of interstitial ion concentrations. This must be viewed in terms of a continuous background of neural activity; thus, the buffering systems are constantly active. This is a relatively straightforward concept to appreciate when referring to extracellular K^+^. A baseline concentration of between 2 and 4 mM must be maintained due to the intimate relationship between K^+^ and the resting membrane potential (both neurons and glia). For example, assuming normal mammalian values of 140 mM for [K^+^]_i_ and 3 mM for [K^+^]_o_, the reversal potential is about −102 mV, but increasing [K^+^]_o_ by only 1 mM depolarises E_K_ by 7.5 mV and doubling [K^+^]_o_ to 6 mM depolarises E_K_ by 18 mV. Such depolarisation of the neural membrane would lead to inactivation of a large number of sodium channels, attenuating excitation and compromising electrical activity [13]. The means by which extracellular K^+^ is predominantly buffered is via astrocytes, which are exclusively permeable to K^+^, with Kir channels and the Na^+^-K^+^-ATPase facilitating uptake of K^+^ [31]. Increasing neuronal activity causes an increase in interstitial K^+^ and a decrease in axonal K^+^ (Figure 7). A simple model would require the redistribution of K^+^ via the axonal uptake of interstitial K^+^. However, buffering is more complex, since some of the metabolic burden is transferred from neurones to astrocytes, which involves intimate neuron to glia metabolic interactions. Rather than simply releasing K^+^ into the local interstitium, the interconnections of astrocytes via gap junctions form syncytia, which allows K^+^ to be redistributed through a much larger volume of tissue.

**Figure 8:**
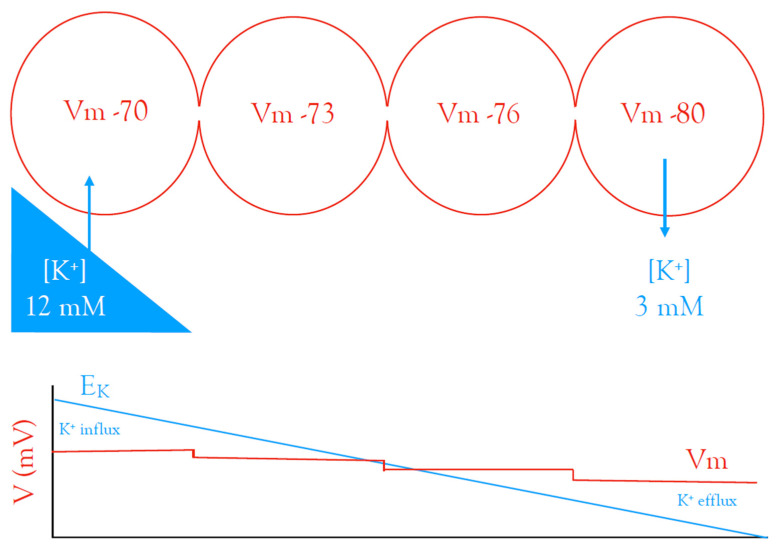
Spatial buffering throughout the astrocyte syncytium. Blue is associated with E_K_ and red with membrane potential (Vm). Localised elevations in [K^+^]_o_ resulting from neuronal activity (left middle—blue triangle) lead to K^+^ influx into nearby astrocytes, causing Vm depolarisation towards E_K_. The Vm does not reach E_K_ as the astrocyte is connected to neighbouring astrocytes, which display a more hyperpolarised Vm. This disparity between Vm versus E_K_ leads to K^+^ influx into astrocytes, which sets up a current through the syncytium, where in more distant astrocytes, the polarity of the difference between Vm and E_K_ switches, leading to K^+^ efflux from the astrocytes (adapted from Ref. [23]).

This process is called spatial buffering and was proposed by Kuffler [23] and was then fine-tuned by Newman [31]. It requires an appreciation of an important but underappreciated aspect of the Nernst equation, namely that when the membrane potential (Vm) is not equal to E_K_, the K^+^ ions will cross the membrane in the direction that drives Vm towards E_K_. Following localised neuronal activity, e.g., intense synaptic activity, local extracellular K^+^ increases, which leads to the depolarisation of the membrane of local astrocytes. This changes E_K_ across the astrocyte membrane, but since the astrocyte is coupled to other astrocytes distant from the region of excitation, they maintain their hyperpolarised Vm. This acts to anchor the astrocyte membrane potential at a more highly hyperpolarised value, which creates a difference between the Vm and E_K_ in those astrocytes, such that extracellular K^+^ enters the astrocyte and spreads throughout the syncytium as a current flow, until it reaches distant astrocytes. The Vm is now greater than E_K_, since the large length constant of these astrocytes means there is a depolarisation in the distant astrocytes, but the E_K_ is maintained at resting values, which leads to K^+^ efflux from these astrocytes in regions distant from activity. In this manner, K^+^ is redistributed from regions of high interstitial concentration to low concentrations (Figure 8).

A similar process to spatial buffering occurs in single cells, such as Muller cells in the retina, where extracellular K^+^ accumulation occurs in the inner and outer plexiform layers in the synaptic regions of the retina. The Muller cells express rectifying K^+^ channels, which facilitates K^+^ uptake. The K^+^ is redistributed to the poles of the cell and released into the vitreous humour and the blood supply at the end feet [65].

Recent data has indicated more detail regarding the mechanism of K^+^ buffering in the rat optic nerve using K^+^-sensitive microelectrodes. The studies revealed a transient increase in interstitial K^+^ in response to high-frequency stimulus that was rapidly buffered towards baseline, immediately on the cessation of the stimulus. A model was proposed in which K^+^ efflux from axons occurred at the onset of high-frequency firing, which accumulated in the interstitial space, leading to a rapid increase of several mM in interstitial K^+^. The time constants of the K^+^ decrease post-stimulus reveals two separate processes. The first process was due to astrocytes buffering interstitial K^+^ using the Na^+^-K^+^-ATPase and Kir channels, whereas the second, slower buffering process involved the Na K pump present on the axonal membrane. Whereas the elevated interstitial K^+^ was the stimulus for the rapid astrocytic buffering, elevated intracellular Na^+^ in the axon was the stimulus for the axonal Na^+^-K^+^-ATPase, leading to Na^+^ efflux, which was coupled to K^+^ influx, to the extent that K^+^ influx was so great its interstitial concentration fell below its baseline value, before slowly relaxing towards the baseline [34]. Thus, a complex story emerges of buffering in central white matter where glial cells and neurons play equitable roles to restore homeostasis rapidly. The importance of the Kir channels expressed in astrocytes in K^+^ buffering, especially at high interstitial K^+^ concentrations, has been demonstrated [35].

## 11. Intracellular Na^+^ Accumulation following Electrical Activity

The ease of use of ion-sensitive glass microelectrodes has favoured studies looking into the effects of high-frequency firing on interstitial K^+^. However, action potentials result in Na^+^ influx into axons; the flux is present throughout the entire nerve in unmyelinated axons and at the nodes in myelinated axons. As we have previously described, the burden of buffering extracellular K^+^, which results from this high-frequency firing, falls to the astrocyte. The situation is different in relation to buffering increases in intercellular Na^+^, since astrocytes play no direct role. In myelinated axons, such as the optic nerve, the Na^+^ channels are expressed in a uniform density at the nodes [104]; thus, the Na^+^ accumulation becomes determined by axon size, with the concentration increase in intracellular Na^+^ a function of axon size [105]. The concentration decreases as the axon size increases, which means that when axons are exposed to high-frequency firing, the intracellular Na^+^ concentration will increase most in the smallest axons. The measurements of the mitochondrial number at the nodes indicate a linear relationship, such that the number of mitochondria increase in proportion to axoplasmic volume [106,107]; thus, the larger axons have proportionally more mitochondria to deal with re-equilibrating a smaller concentration of Na^+^ ions. This is apparent given the decrease in the action potential amplitude of small axons exposed to high-frequency firing, when compared to larger axons. It is extremely difficult to measure intracellular Na^+^ concentrations in nodes and impossible to dissect this down to axons of different diameters; therefore, investigations of this type are predictably based on computational modelling. These models rely on previously published equations relating to the Hodgkin and Huxley model, the Nernst equation and the surface-area-to-volume relationships [105,106,107]. These studies are complicated, as there are multiple factors present that dictate the effect on compartmental ion concentrations as a result of high-frequency firing. The first is the effect of axon size. In the optic nerve, there are relatively few axons greater than 0.7 μm in diameter [108], begging the question as to why are they present? It is proposed they conduct important information at high frequency, since larger axons conduct a larger bit size than smaller axons [107]. The triple peaked profile of the stimulus-evoked compound action potential in the mouse optic nerve is an obvious indication of the presence of axons of different size, with the larger axons being the least numerous in this tissue diameter [108]. Calculations have proposed that the smallest axons in the mouse optic nerve can conduct at a maximum frequency of 5 Hz [106]. Any frequency above this leads to a reduction in the amplitude of the CAP peak, which is contributed to by the smallest axons.

## 12. Conclusions

The use of biosensors, specifically K^+^-sensitive microelectrodes, has provided a wealth of information relating to activity-dependent and pathological changes in interstitial [K^+^]. As a result of activity, K^+^ leaves neural elements via voltage-dependent K^+^ channels, as described by Hodgkin and Huxley. In addition, during metabolic insult when ATP supply is limited, the Na^+^-K^+^-ATPase is starved of fuel, leading to the dissipation of trans-membrane gradients, ultimately resulting in interstitial K^+^ accumulation. Since neuronal membranes are predominantly permeable to K^+^ at rest, this leads to membrane depolarisation and the inactivation of Na^+^ channels, which attenuates activity. There are powerful buffering mechanisms to maintain homeostasis and restore interstitial [K^+^] to its baseline levels. These include the ability of astrocyte to take up K^+^ via the Na^+^-K^+^-ATPase and Kir channels, but axon membranes use the activity-dependent increases in axonal [Na^+^] to activate the Na^+^-K^+^-ATPase pump, an alternate mechanism for buffering interstitial K^+^.

## Data Availability

The data presented in this study are available on request from the corresponding author.
